# Supplementary material for: Humanized L184Q Mutated Surfactant Protein C Gene Alters Alveolar Type 2 Epithelial Cell Fate
Source: Int J Mol Sci. 2024 Aug 9;25(16):8723. doi: 10.3390/ijms25168723 (PMC11354303; doi:10.3390/ijms25168723)

# Humanized L184Q Mutated Surfactant Protein C Gene Alters Alveolar Type 2 Epithelial Cell Fate

Krishan G Jain [1], Yang Liu [1], Runzhen Zhao [1], Preeti J Muire [2,3,4], Jiwang Zhang [5,6], Qun Sophia Zang [1,2] and Hong-Long Ji [1,2]

**Supplementary data**

**Figure S1.** Full size western blots.

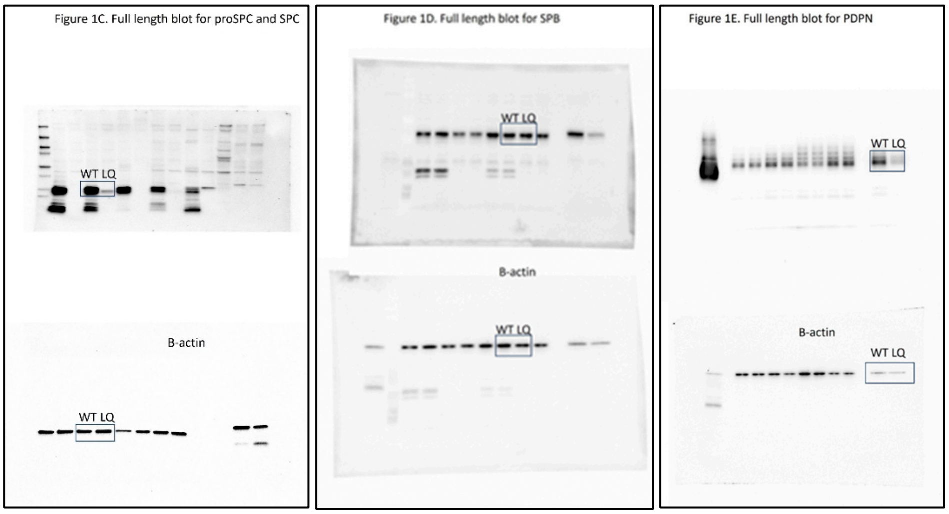

**Figure S2.** Full size western blots.

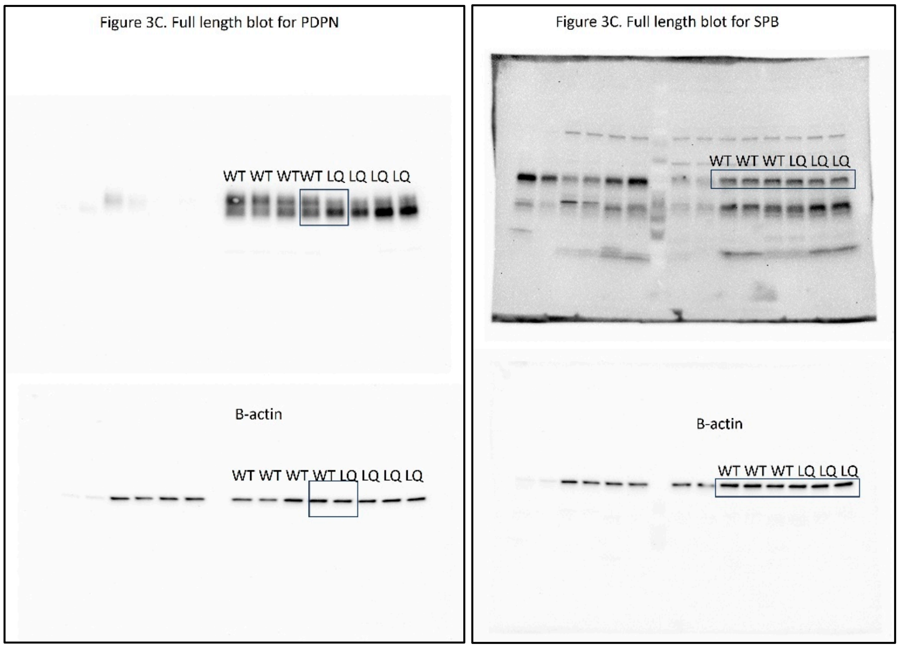


**Figure S3.** Full size Western blot.

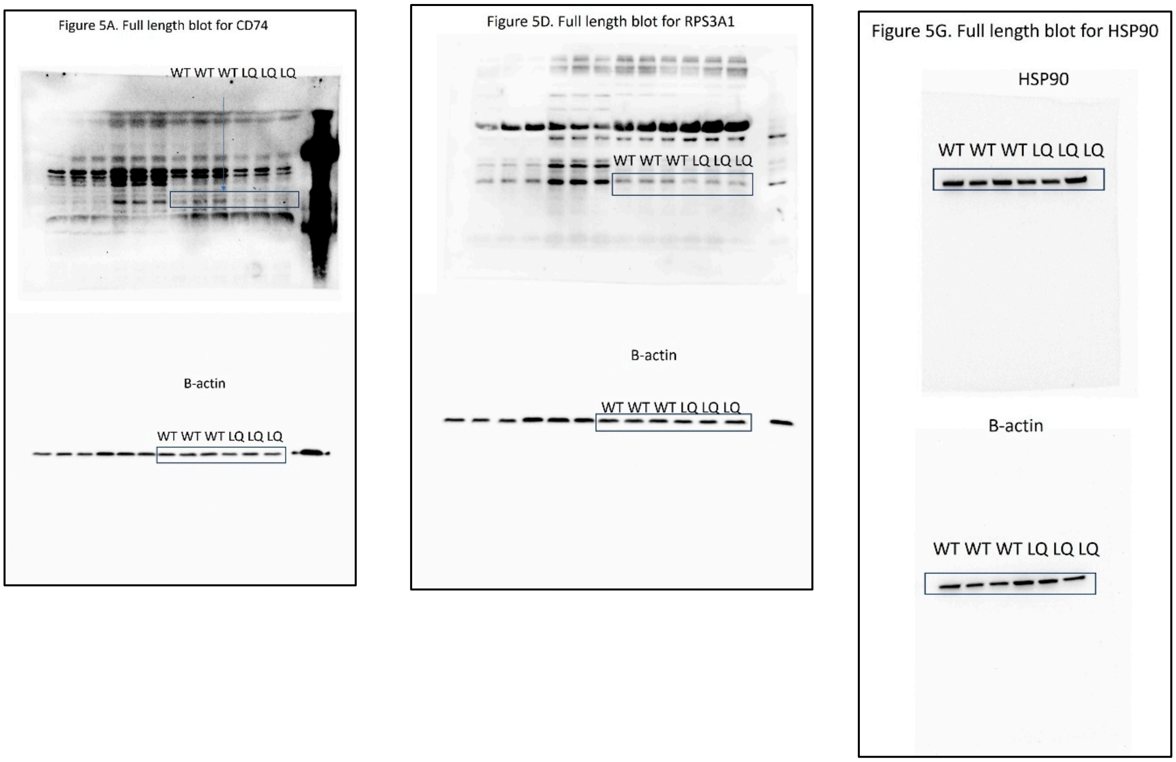

**Figure S4.** Purity of FACS sorted AT2 cells.

We performed immunofluorescent staining of FACS-sorted cells, which showed 98% SPC + AT2 cells. Here is an image of immunofluorescence staining for the purity of AT2 isolation. FACS sorted AT2 (WT) cells were cytospun on glass slides and labeled with SPC antibody and DAPI. Scale bar 100μm

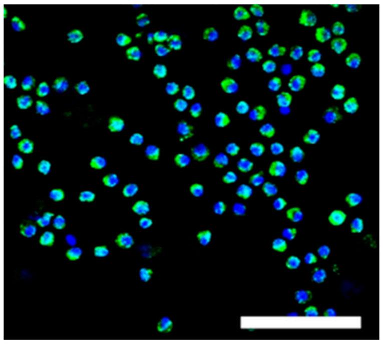

Supplement: Supplementary file 1 [file ijms-25-08723-s001.zip › ijms-3080215-supplementary.pdf]
